# Classifying fibromyalgia patients according to severity: the combined index of severity in fibromyalgia

**DOI:** 10.1007/s00296-014-3029-8

**Published:** 2014-05-04

**Authors:** J. Rivera, M. A. Vallejo, M. Offenbächer

**Affiliations:** 1 Unidad de Reumatología, Instituto Provincial de Rehabilitación, Hospital General Universitario Gregorio Marañón, Francisco Silvela 40, 28028 Madrid, Spain; 2 Departamento de Psicología de la Personalidad, Evaluación y Tratamientos Psicológicos, UNED, Madrid, Spain; 3 Generation Research Program, Human Science Center, University of Munich, Bad Tölz, Germany

**Keywords:** Fibromyalgia, Severity, Outcome assessment, Questionnaires, Instruments, Subgroups

## Abstract

The aim of this study was to establish the cutoff points in the Combined Index of Fibromyalgia Severity (ICAF) questionnaire which allow classification of patients by severity and to evaluate its application in the clinical practice. The cutoff points were calculated using the area under the ROC curve in two cohorts of patients. Three visits, basal, fourth month and 15th month, were considered. The external criterion for grading severity was the number of drugs consumed by the patient. Sequential changes were calculated and compared. Correlations with drug consumption and comparisons of severity between patients with different types of coping were also calculated. Correlation between the number of drugs and the ICAF total score was significant. Three cutoff points were established: absence of Fibromyalgia (FM), <34; mild, 34–41; moderate, 41–50 and severe, >50, with the following distribution of severity: absence in 0.4 %, mild in 18.7 %, moderate in 32.5 % and severe in 48.4 % of the patients. There were significant differences between groups. The treatment under daily clinical conditions showed a significant improvement of the patients which was maintained at the end of follow-up. There was a 17 % reduction in the severe category. The patients with more passive coping factor showed highest punctuations in the remaining scores and were more prevalent in the severe category. The patients with a predominance of the emotional factor showed a better response at the end of follow-up. The established cutoff points allow the classification of FM patients by severity, to know the prognostic and to predict the response to the treatment.

## Introduction

Fibromyalgia (FM) is a syndrome with multiple clinical manifestations from various organs and systems and not only a clinical picture of chronic pain. Therefore, a proper evaluation of FM must include not only pain but its most prevalent clinical manifestations, as has recently been recognized with the new diagnostic criteria based on clinical symptoms [1].

The evaluation of the severity in FM patients is still difficult since there is no gold standard outcome measure with which to compare the clinical manifestations of this syndrome. Nowadays, multiple questionnaires and other instruments are being used to measure the severity of each FM symptom [2].

The Fibromyalgia Impact Questionnaire (FIQ) [3] was developed in 1991 to measure the impact of FM and it soon became the most generalized tool for evaluating FM patients. FIQ offers a total score which allows to evaluate the impact of FM in patient life. Later, and following the criterion of pain intensity alone, cutoff points were calculated, allowing a classification of FM severity into mild, moderate and severe [4]. However, a classification of patient severity following the unique criterion of pain shows a clear bias since most of them are classified as severe.

Recently, we have developed and validated the Combined Index of Fibromyalgia Severity (ICAF, acronym for Índice Combinado de Afectación en Fibromialgia) [5, 6], a new tool for evaluating FM severity based on its most prevalent clinical manifestations. ICAF shows a total score of global severity where the higher the total score, the more severe are the disease and the consequences in patient life. ICAF questionnaire may also provide information about emotional, physical and coping aspects of the patient, but a classification of patient by severity degrees has not been developed so far.

The present study has been designed to establish the cutoff points in the total score of ICAF questionnaire based on the hypothesis that the number of drugs for treating FM patients may be used as a criterion of severity. Subsequently, the calculated cutoff points were applied in a cohort of FM patients under daily clinical practice conditions to determine the degree of disability, the best treatment options and the evolution of the patients.

## Patients and methods

### Patients

In this work, two cohorts of patients were studied.

#### Cohort 1

In order to determine the cutoff points, we used the original cohort of patients from the ICAF study [6]. This cohort consisted of 232 patients (F: 228 and M: 4) fulfilling ACR 1990 criteria for FM [7], with a mean age of 47.73 ± 8.61 years. All patients were attended by FM specialized clinicians from all around the country. Informed consent was signed by patients and the protocol was approved by the Ethic and Clinical Investigation Committee of Hospital Universitario Gregorio Marañón of Madrid. A control group of 110 healthy people was also studied.

#### Cohort 2

The newly established cutoff points were finally evaluated in a second cohort of patients consisting of 246 FM consecutive patients (F: 233 and M: 13) with a mean age of 48.5 ± 9.8 years. All these patients were attended in a specialized clinic under daily clinical practice conditions. The ICAF questionnaire was used for evaluating these patients in consecutive visits, at the beginning of treatment (V1), at fourth month (V2) and at 15th month (V3) of follow-up.

### ICAF questionnaire

Briefly, the ICAF is a self-administered questionnaire composed of 59 items about the most common clinical manifestations of FM [5]. It calculates a total score in such a way that a higher score indicates a more severe disease. It also offers four indices: emotional, physical, active coping and passive coping. The emotional factor stresses the role of emotional aspects such as anxiety and depression; the physical factor evaluates pain, fatigue, sleep quality and functional capacity; the active and passive coping factors include different coping strategies by which patients cope with the disease. Calculated direct scores are transformed into a T scale with a mean of 50 and standard deviation of 10 related to the sample studied.

Similar to total score, higher scores of factors indicate more severity with the exception of active coping factor in which higher scores indicates a better way to cope with the disease.

In Vallejo et al. [5], the reference percentiles to locate the patient with respect to the sample are shown, indicating that severity is always related to the sample studied.

The weight of each factor in the calculation of the total score is different. The emotional factor constitutes 66 % of the total score while physical factor is about 23 %. Coping factors have a relative small weight with a 6 % for each of them [5].

### Measure of severity

Previously, in the construction of the ICAF, scores of the different factors and total punctuation were correlated with external criteria of FM severity such as the presence of trigger points, the results of the six-minute walk test, or the labor situation, among others [5]. In this work, we have used a different external criterion of severity: the number of prescribed drugs a patient is taking for treating the disease. The working hypothesis in this study has been that the number of drugs acting over nervous system (NS) is related to the severity of the disease in the sense that a higher number of drugs are needed to treat a more severe disease.

The severity based on the number of drugs consumed by the patient has been established with the following criterion:

- Mild: Consumption of NSAID or analgesics only.
- Moderate: Consumption of one or two drugs acting over NS. The most common combination is an antidepressant together with a benzodiazepine or hypnotic drug.
- Severe: Consumption of three or more drugs acting over NS. New types of drugs are introduced or different antidepressants or benzodiazepines are added to the previous treatment.

A correlation between the ICAF total score and its factors and drug consumption was also studied.

The cutoff points based on pain intensity established by Bennett et al. [4] were used for calculating the severity of the disease in the FIQ.

### Statistical analysis

To establish cutoff points in the ICAF, the areas under the ROC curves were calculated in cohort 1 of patients. For each cutoff point, the best equilibrium between specificity and sensitivity was considered in the context of general data.

Correlation between total score and different factors with drug consumption was calculated with the Pearson correlation test.

Chi-square test was used to compare severity between patients with different types of coping.

To compare sequential changes in total score and its factors along the different visits, an analysis of variance with repetitive measures was used. Bonferroni correction was used to compare pairs.

Statistical analysis was conducted using SPSS version 19. A *p* value <0.05 was accepted for statistical significance.

## Results

In cohort 1 of patients, the mean FIQ score was 71.28 ± 15.34. The distribution of the severity calculated with the FIQ was mild in 2.6 %, moderate in 18.5 % and severe in 78.9 % of the patients.

Correlation between drug consumption and the ICAF total score was significant (*r* = 0.323; *p* < 0.001), especially for drugs acting over NS (*r* = 0.384; *p* < 0.001) with the exclusion of analgesics and NSAID. Table 1 shows the increase in the ICAF total score in relation to the increase in the number of drugs acting over NS consumed by the patients. Classification of patients by severity related to the number of drugs consumed is also shown in Table 1. There exists a significant increase in the ICAF total score depending on severity (mild vs. moderate, *p* < 0.03; moderate vs. severe, *p* < 0.001).

**Table 1:** Classification of severity, number of drugs acting over nervous system consumed by the patients and ICAF total score

	No. of drugs^a^	No. of patients (%)	ICAF total score Mean (SD)	No. of patients (%) in the category	ICAF total score Mean (SD) in the category
Mild	0	61 (26.3)	47.23 (7.86)	61 (26.3)	47.23 (7.86)
Moderate	1	73 (31.5)	48.97 (8.94)	124 (53.5)	50.15 (8.81)
	2	51 (22.0)	51.84 (8.42)		
Severe	3	29 (12.5)	56.62 (9.95)	47 (20.2)	57.52 (10.53)
	4	14 (6.0)	58.70 (12.98)		
	5	4 (1.7)	59.97 (4.59)		
Total		232 (100)		232 (100)	

^a^Includes antidepressants, benzodiazepines, hypnotics, anticonvulsants, antipsychotic and antihistaminic, with the exclusion of analgesics (any type) and NSAID

**Fig. 1:**
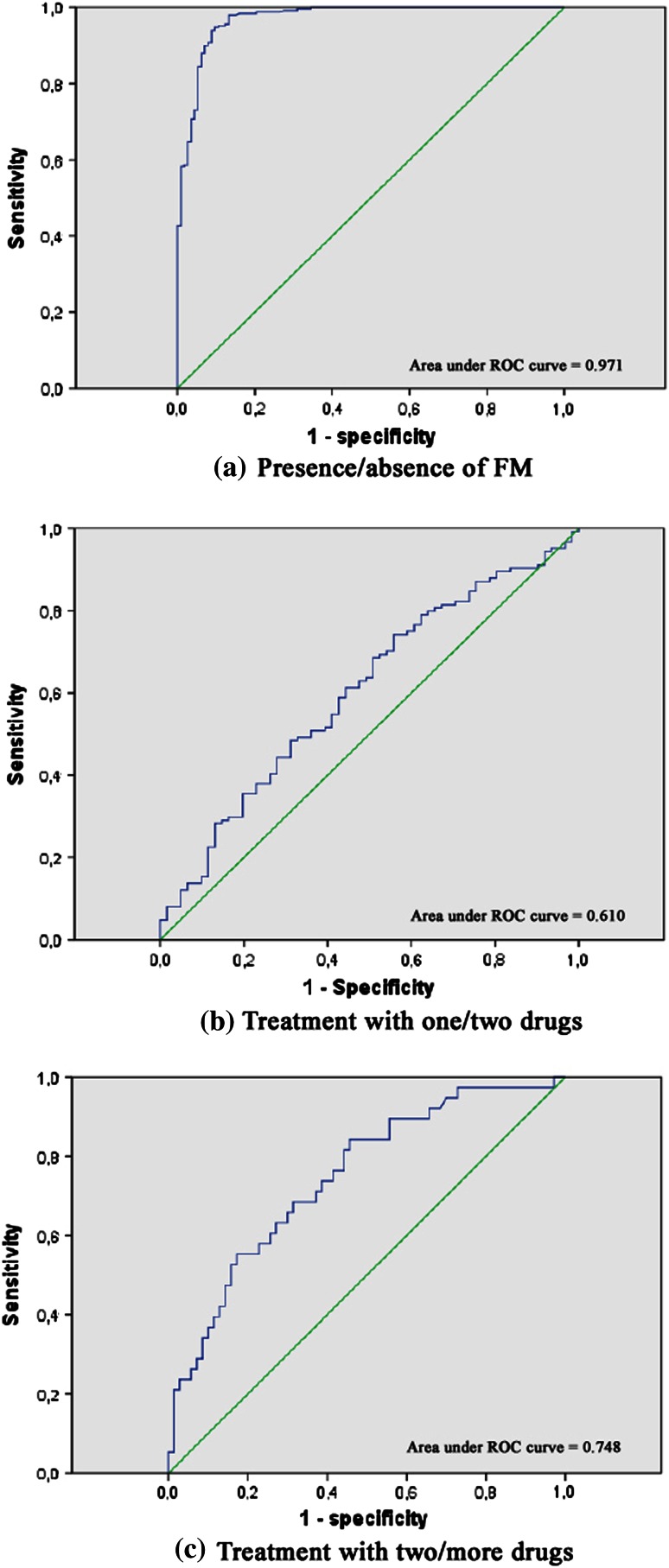
ROC curves

The first cutoff point was calculated in the total score to differentiate patients and controls. The cutoff point was established in 34, with a sensitivity of 0.996 and specificity of 0.727 (Fig. 1a).

To calculate the second cutoff point to differentiate absence of FM from mild severe disease, the considered option was to enhance specificity. This cutoff point was established in 41 with a sensitivity of 0.944 and a specificity of 0.903 (Fig. 1b).

The third cutoff point to differentiate between mild and moderate severity was established in 50, with a sensitivity of 0.581 and a specificity of 0.574 (Fig. 1c).

Taking into account these cutoff points, in the ICAF total score, the severity of patients with FM may be divided into:

Absence of: FM <34
Mild: 34–41
Moderate: 41–50
Severe: >50

The distribution of patients in cohort 1 of patients was: absence of FM in 3.4 %, mild in 10.8 %, moderate in 35.3 % and severe in 50.4 % of the patients.

In cohort 2, the application of the previously calculated cutoff points at V1 showed the following distribution: absence of FM in 0.4 %, mild severity in 18.7 %, moderate severity in 32.5 % and severe severity 48.4 % of the patients (Table 2). There exist significant differences between groups in all total and factor scores, with the exception of the passive coping factor, which is similar in moderate and severe categories.

**Table 2:** Classification by severity in cohort 2 of patients using cutoff points at V1

	Severity		
	Mild	Moderate	Severe
	No. of patients	No. of patients	No. of patients
	47 (19.1 %)	80 (32.5 %)	119 (48.4 %)
	Mean (SD)	Mean (SD)	Mean (SD)
ICAF total	36.47 (3.61)	46.29 (2.96)	60.21 (6.05)
Emotional	38.53 (4.57)	45.83 (4.95)	60.17 (7.12)
Physical	40.92 (8.57)	50.34 (6.95)	58.10 (6.15)
Active coping	60.61 (5.63)	54.93 (8.53)	46.13 (9.73)
Passive coping	45.06 (8.71)	50.32 10.45)	52.50 (11.86)

Total and factor scores at V1

At visit V2, the treatment under daily clinical practice conditions showed a significant improvement of the patients (Table 3). The ANOVA performed with the three visits showed significant effects in the ICAF total score [*F*(2,82) = 12.49, *p* < 0.001], emotional factor [*F*(2,82) = 15.47, *p* < 0.001] and physical factor [*F*(2,82) = 10.15, *p* < 0.001], while there were no significant effects in the active coping factor [*F*(2,82) = 0.72, *p* < 0.49] or the passive coping factor [*F*(2,82) = 0.09, *p* < 0.91]. The significant improvement obtained at V2 is also maintained at V3 15 months later of V1. Means and multiple comparisons are shown in Table 3.

**Table 3:** Total ICAF and factors score along visits

	V1 Mean (SD)	V2 Mean (SD)	V1–V2 diff.	CI 95 %	V3 Mean (SD)	V1–V3 diff.	CI 95 %
ICAF total	51.13 (10.12)	46.73 (10.39)	4.40*	1.98–6.82	46.07 (11.03)	5.06*	2.03–8.09
Emotional	52.86 (10.59)	48.18 (9.85)	4.68*	2.26–7.09	47.40 (10.05)	5.46*	2.52–8.39
Physical	51.14 (9.11)	45.96 (12.70)	5.18*	2.41–7.94	46.33 (12.49)	4.81*	1.71–7.90
Active coping	53.34 (10.09)	53.61 (9.75)	−0.27	−2.77–2.24	54.46 (9.40)	−1.12	−3.47–1.23
Passive coping	50.70 (11.56)	50.50 (11.26)	0.20	−2.20–2.60	50.24 (11.70)	0.46	−2.49–3.41

* *p* < 0.001, Bonferroni

The favorable effect of the treatment throughout time produced an evolution of the severity with an increase in the less severe categories, as show in Table 4. At V3, there was a 17 % reduction in the severe category with a similar increase in the less severe categories (absence plus mild).

**Table 4:** Evolution of the severity categories along visits

	V1	V2	V3
	No. of patients (%)	No. of patients (%)	No. of patients (%)
Absence of FM	1 (0.4)	18 (11.3)	12 (13.3)
Mild	46 (18.7)	23 (14.5)	20 (22.2)
Moderate	80 (32.5)	76 (47.8)	30 (33.3)
Severe	119 (48.4)	42 (26.4)	28 (31.1)

In the analysis of coping predominance, patients with more passive coping factor score, i.e., those who have a higher score in the passive coping than in the active coping factor, showed highest scores in total, emotional and physical factors, and may be considered to be worse. These patients consume more drugs, specially benzodiazepines and antidepressants. The severity in V1 also showed a significant prevalence of the severe category among the patients with more passive coping strategies (*χ* ^2^ (3) = 70.30, *p* < 0.001) (Table 5).

**Table 5:** Severity in patients classified by coping strategy predominance at V1 and its evolution at V2

	Coping predominance			
	V1		V2	
	Active	Passive	Active	Passive
	No. of patients (%)	No. of patients (%)	No. of patients (%)	No. of patients (%)
Absence of FM	1 (0.7)	0	14 (15.4 %)	4 (5.9 %)
Mild	45 (33.6)	1 (0.9)	16 (17.6 %)	7 (10.3 %)
Moderate	53 (39.6)	27 (24.1)	44 (48.4 %)	32 (47.1 %)
Severe	35 (26.1)	84 (75.0)	17 (18.7 %)	25 (36.8 %)

At V2, the treatment showed a good response in both groups of patients with a predominance of the active or the passive coping. However, at V2, the analysis of severity still showed a significantly worse situation in patients with a predominance of passive coping strategies (*χ* ^2^ (3) = 9.36, *p* = 0.025) (Table 5).

The response to the treatment in patients with a predominance of the emotional factor or the physical factor was similar, with no statistical differences between them at V2. However, in the group with a higher score in the physical factor than in the emotional factor, at V3, there was no statistical difference with respect to V1, which indicates a lack of response to the treatment. By contrast, the group of patients with a higher score in the emotional factor still maintained a significant response to the treatment at V3.

## Discussion

The ICAF is a self-administered questionnaire designed for the evaluation of multidimensional aspects of FM patients [5]. As previously mentioned, it already contains a severity reference by assigning the corresponding place in the original distribution of patients studied for the development of the instrument. This explains the normalized punctuations (*T* score) used in this questionnaire. Also in the development of the ICAF, external criteria such as labor status, presence of trigger points and six-minute walk test were employed for the validation of the punctuation distribution [5].

In the current work, an additional criterion has been considered: measuring global severity by the number of consumed drugs since it better reflects the overall situation of the patient compared with the severity based on pain intensity.

It is known that in addition to generalized pain in FM there exist other relevant clinical manifestations such as sleep disorders, fatigue, depression, or anxiety. For this reason, it is very common that patients with FM consume other drugs acting over NS for treating all those clinical manifestations, in addition to analgesics and NSAID to alleviate pain.

In this work, we have started with the hypothesis that the higher the severity of the disease, the higher number of drugs consumed by the patient. This fact has been previously confirmed by Sánchez et al. [8] who demonstrated that economical costs associated with drug treatment in FM increase by comorbidity rather than with drugs to alleviate pain. We have considered that our point of view may be questionable, but we also believe that this approach may help clarify the always difficult aspect of quantifying severity in FM patients.

In our cohort of patients, distribution of severity after applying ICAF calculated cutoff points was 14, 35 and 50 % for mild, moderate and severe forms, respectively, whereas distribution of the severity following the FIQ cutoff points yielded 3, 18 and 79 %, respectively. As it can be seen, the distribution is more normal using the ICAF cutoff points with a lesser number of severe forms and a higher frequency of mild ones. In the original work by Bennett et al. [4] where cutoff points were established for the FIQ, the mild forms were only observed in 6 % of the patients.

Our results confirm the initial working hypothesis and the calculated cutoff points allow the classification of patients in a more practical way than other existing questionnaires. Classification of the disease in terms of severity also allows to establish an overall prognosis. In this work, 77 % of patients classified as mild still remained with the same degree of severity, whereas 50 % still continued to be classified as severe 1 year later.

To classify patients by severity also has several implications in the field of therapeutic decisions and economic costs of resource utilization. For those patients classified as severe, it seems logical to intensify treatment specially by using other therapeutic approaches such as physical exercise programs as well as psychological therapeutic modalities. Incorporating psychoeducational resources in primary care may improve therapeutic response and reduce the probability of impairment in these patients [9].

The ICAF questionnaire also provides interesting information through the different factors in which it is composed. The emotional factor provides the most factorial component with respect to the total score and patients with higher punctuation in the emotional factor also have a higher total score, as it is shown in this work. However, these patients also have a good response to the treatment and they would be the better candidates for drug treatment, especially antidepressants.

A high punctuation in the physical factor is associated with a long-term poorer response, as it is shown in our results. In this group are included those patients with higher degrees of fatigue, closer to chronic fatigue syndrome, and it is known that these patients have a poor response to pharmacological treatments, especially to antidepressants [10]. In this group of patients, exercise programs should be emphasized and more sophisticated physiotherapy programs with well-elaborated adherence strategies may be the best therapeutic approach.

Although both coping factors only add a small percentage to the ICAF total score, the evaluation of coping strategies is very useful [11]. Indeed, as shown by our results, coping factor scores are determinant to determine the severity of the disease. Patients with high passive coping scores have higher total scores, consume more drugs and also have a poorer prognostic. Intensifying drug therapy in these patients does not seem to be the best strategy since they are already taking more drugs than the rest of the patients and that does not work properly for them. They need a personalized psychotherapeutic intervention or a guided psychological therapy to improve coping with the disease and to reduce the probability of impairment. Alternatively, psychological group therapy or via the Internet has been also shown to be useful [12, 13].

In comparison with other FM global outcome indexes, the ICAF shows some strengths and some disadvantages. As a clear strength, it is remarkable that the ICAF has been confronted with external criteria not coming from the patient self-report, in contrast with other tools and questionnaires such as CODI [14], CRSFS [15] or FiRST [16], in which no external criteria were used for its construction. Another strength is the presence of the two coping factors, because good coping strategies are very important in the favorable outcome in chronic diseases, and the aforementioned questionnaires do not specifically consider such aspects.

It is difficult to cover all principal clinical aspects of FM with a short questionnaire. Indeed, the ICAF contains 59 items, while CODI has 26 [14], CRSFS has 20 [15] and FiRST has 6 items [16]. Even considering that shortness is an important aspect of a questionnaire, it could not be at the expense of eliminating basic aspects in the evaluation of an FM patient, as it occurs with emotional aspects in the FiRST questionnaire [16]. On the other hand, a more complete questionnaire may contribute to clarify some believes that general practitioners have about FM [17].

Outcomes measures in FM still remain controversial. Recently, the OMERACT has elaborated a report based on experts’ opinion about the most important outcome measures that must be evaluated in patients with FM [18]. However, an online interview with patients [19] shows that there exist some highly valued variables by the patients that are not included in the experts inform of OMERACT. A possible explanation of this divergence between patients and experts may be that for some aspects such as stiffness or cognitive dysfunction, highly evaluated by the patients, there are no effective instruments of measurement, and for this reason, the experts do not include them.

In recent clinical trials with drugs for treating FM, the most common outcome measure is pain [2]. However, pain is just an aspect of the disease and in some patients not even the most important clinical manifestation. When a clinical trial is designed, if this consideration is not taken into account, there may be different populations of FM patients with different responses to the same treatment. So, a favorable response in the outcome of pain may be misinterpreted if the intervention does not improve some of the aspects of FM.

The strong point of this study is that results about classification of severity obtained in a multicentric cohort of patients with FM have been well reproduced in a different cohort of patients in daily clinical practice showing the same useful information.

The principal limitation of this study lies in that the ICAF has not been proven in clinical trials and has not been compared with other tools. However, if ICAF has shown to be sensible to the changes that occur in daily practice conditions—both in short and in longer periods of time—the response will probably be similar under clinical trial conditions.

In addition, in this study, we have not established a comparison with an untreated control group, and for this reason, it is not possible to determine whether changes observed throughout time are due to the treatment itself.

In conclusion, the ICAF has shown to be an excellent outcome measure, which allows the classification of FM patients by severity in a very practical way, to determine the prognostic of the disease and to predict the response to the treatment through its different component factors. These are all important issues which impact positively on usual clinical practice with FM patients, and provide invaluable information which is now difficult and time consuming to obtain.
